# Supplementary material for: Structure of the transcription open complex of distinct σI factors
Source: Nat Commun. 2023 Oct 13;14:6455. doi: 10.1038/s41467-023-41796-4 (PMC10575876; doi:10.1038/s41467-023-41796-4)
Supplement: Supplementary file 1 — Supplementary Information [file 41467_2023_41796_MOESM1_ESM.pdf]

Supplementary Materials for

Structure of the transcription open complex of distinct σ^I factors

Jie Li [1, 2, 3, 4, 5, 6, #], Haonan Zhang [6, 7, #], Dongyu Li [6, 7], Ya-Jun Liu [1, 2, 3, 4, 5, 6], Edward A. Bayer [8, 9], Qiu Cui [1, 2, 3, 4, 5, 6], Yingang Feng [1, 2, 3, 4, 5, 6, *], Ping Zhu [6, 7, *]

[1] CAS Key Laboratory of Biofuels, Qingdao Institute of Bioenergy and Bioprocess Technology, Chinese Academy of Sciences, Qingdao, Shandong, 266101, China

[2] Shandong Provincial Key Laboratory of Synthetic Biology, Qingdao Institute of Bioenergy and Bioprocess Technology, Chinese Academy of Sciences, Qingdao, Shandong, 266101, China

[3] Shandong Engineering Laboratory of Single Cell Oil, Qingdao Institute of Bioenergy and Bioprocess Technology, Chinese Academy of Sciences, Qingdao, Shandong 266101, China

[4] Shandong Energy Institute, Qingdao, Shandong, 266101, China

[5] Qingdao New Energy Shandong Laboratory, Qingdao, Shandong, 266101, China

[6] University of Chinese Academy of Sciences, Beijing 100049, China

[7] National Laboratory of Biomacromolecules, CAS Center for Excellence in Biomacromolecules, Institute of Biophysics, Chinese Academy of Sciences, Beijing 100101, China.

[8] Department of Biomolecular Sciences, The Weizmann Institute of Science, Rehovot 7610001, Israel.

[9] Department of Life Sciences and the National Institute for Biotechnology in the Negev, Ben-Gurion University of the Negev, Beer-Sheva 8499000, Israel.

[#] These authors contributed equally to this work.

* Corresponding authors. E-mail: fengyg@qibebt.ac.cn (Yingang Feng) and zhup@ibp.ac.cn (Ping Zhu)

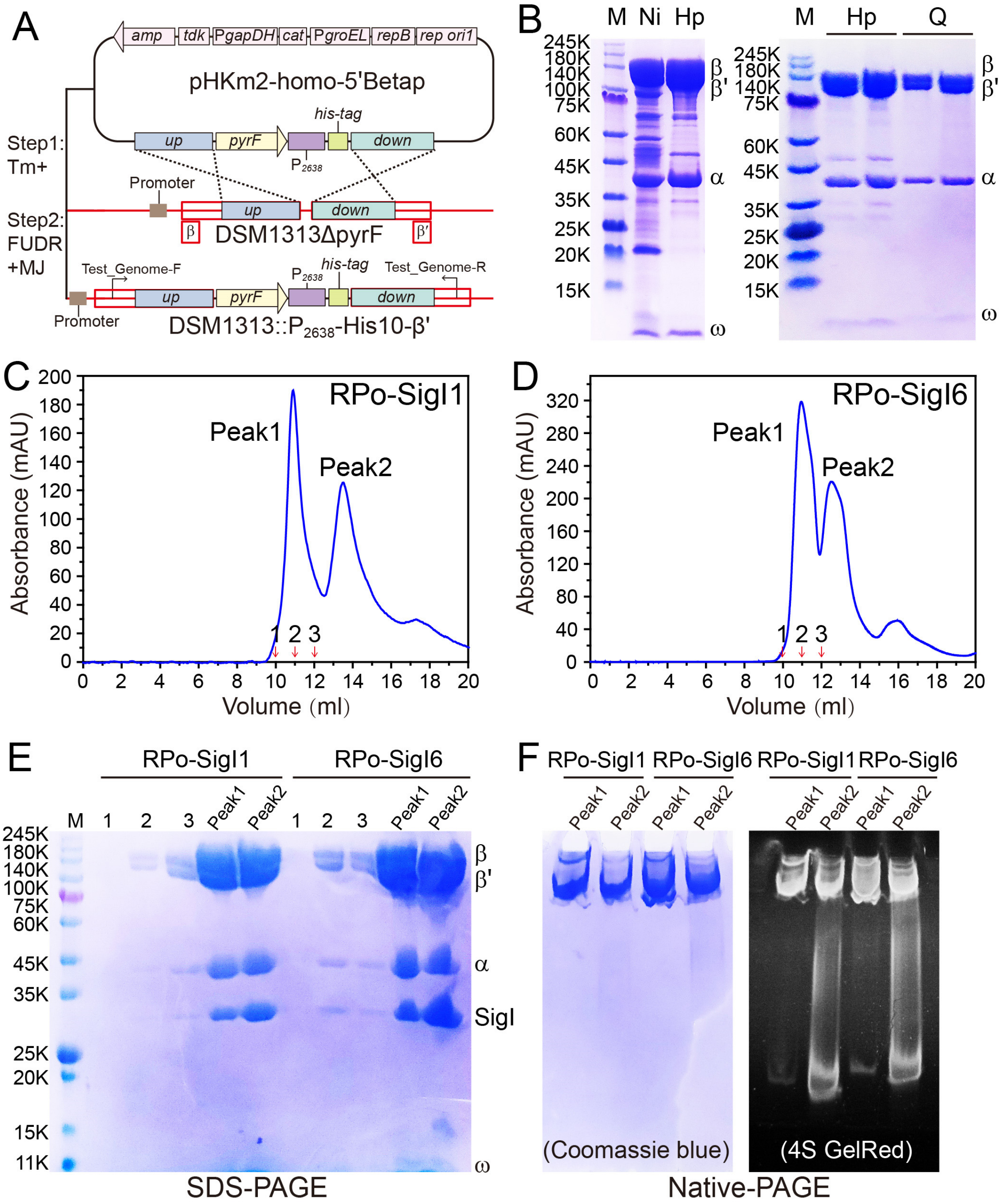

Fig. S1 Reconstruction and purification of *Clostridium thermocellum* RNAP-SigI-promoter open complexes RPo-SigI1 and RPo-SigI6. (A) Schematic diagram showing genetic modification of the β' subunit (*clo1313_0314*) for adding a constitutive promoter and an N-terminal His×10-tag. (B) Tricine SDS-PAGE analysis for the purification of the RNAP core enzyme from *C. thermocellum* DSM1313::His$_{10}$-β'. Lanes Ni, Hp, and Q comprise samples, following purification by Ni^{2+}-affinity chromatography, heparin column, and Source Q column, respectively. Lane M, the molecular weight marker. (C-D) Gel-filtration elution profiles of reconstituted RPo-SigI1 (C) and RPo-SigI6 (D). Peak1 contains the reconstituted RPo complexes and Peak2 includes the excess proteins and nucleic acids that cannot be reconstituted in the RPo complex. (E) SDS-PAGE analysis of the gel-filtration elution peaks. Lanes 1-3 are

the eluted fractions as indicated in (C) and (D) by red arrows. Lanes Peak1 and Peak2 are the concentrated Peak1 and Peak2 fractions in the gel-filtration. (F) Native-PAGE analysis of Peak1 and Peak2 fractions eluted in the gel-filtration. The gels were stained by Coomassie blue and 4S GelRed for visualization of protein and DNA, respectively.

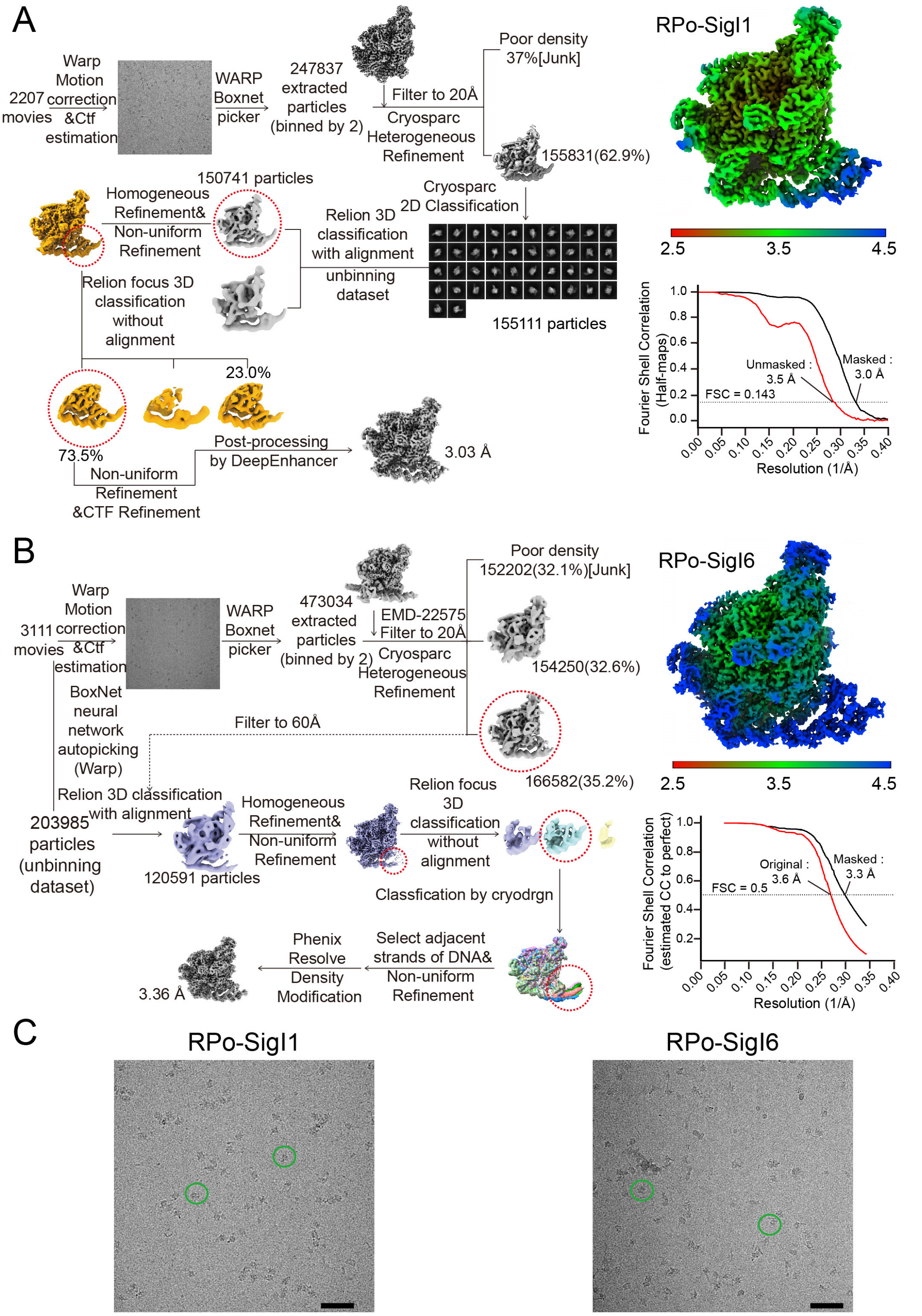

Fig. S2 Procedures for CryoEM structure determination of RPo-SigI1 (A) and RPo-SigI6 (B). (C) Representative cryo-EM micrographs of RPo-SigI1 (left) and RPo-SigI6 (right) complexes. Green circles indicate examples of individual particles. The scale bar is 50 nm.

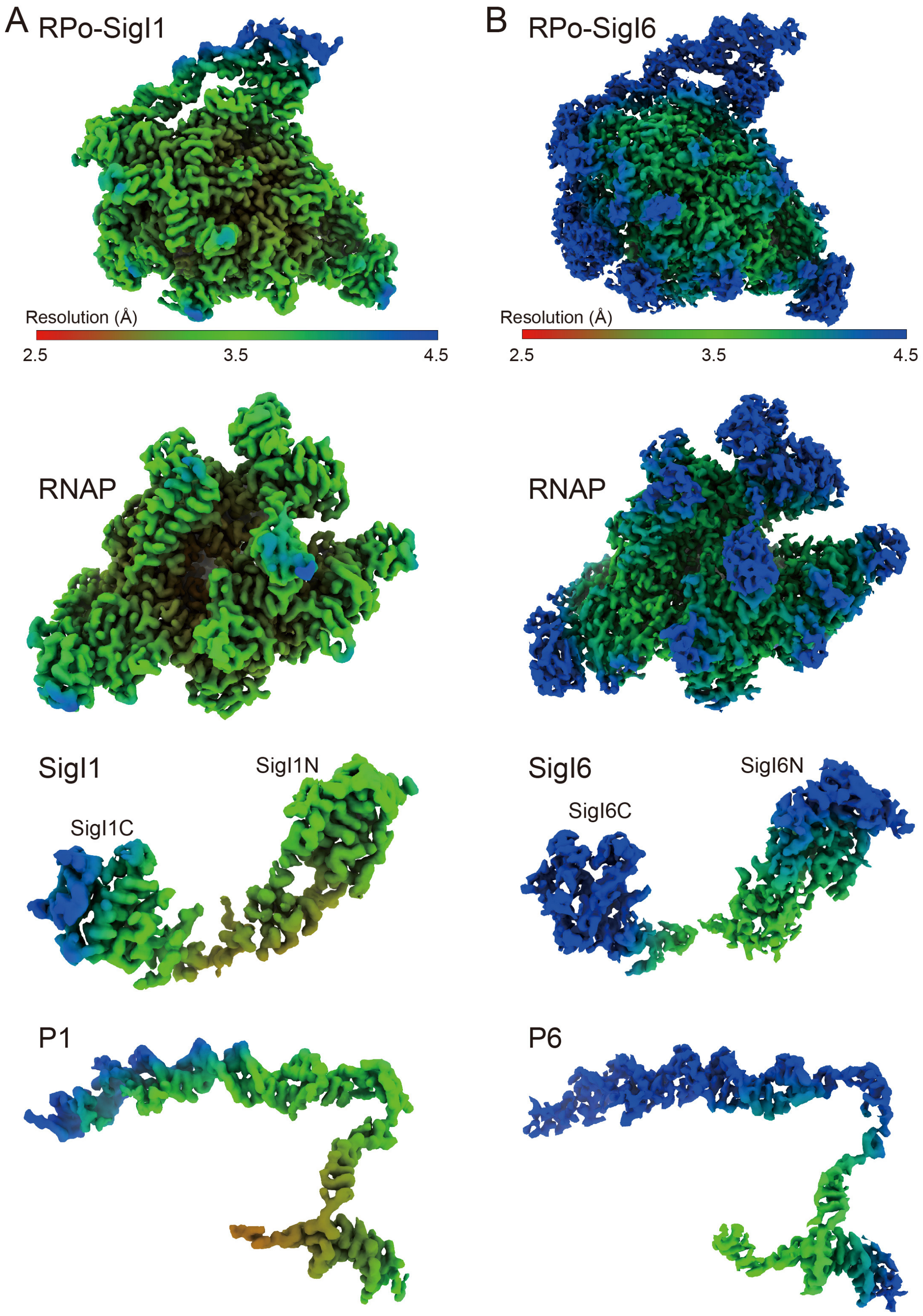

Fig. S3 Local resolution maps for RPo-SigI. The local resolution maps of the whole complex and individual parts for RPo-SigI1 (A) and RPo-SigI6 (B) are shown with red-green-blue gradient colours for resolution ranges between 2.5-3.5-4.5 Å.

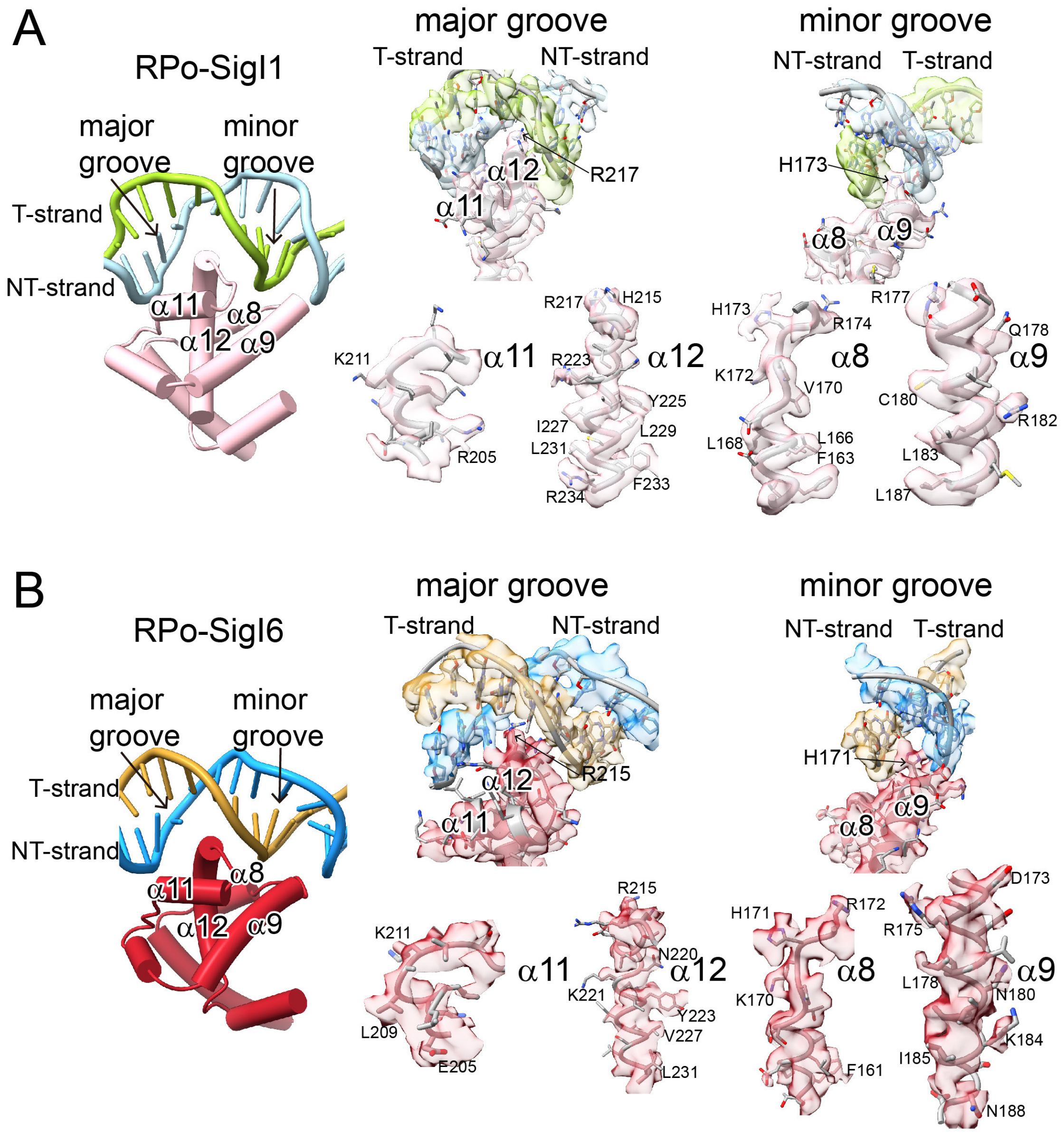

Fig. S4 Density maps of SigI1C/-35 DNA (A) and SigI6C/-35 DNA (B).

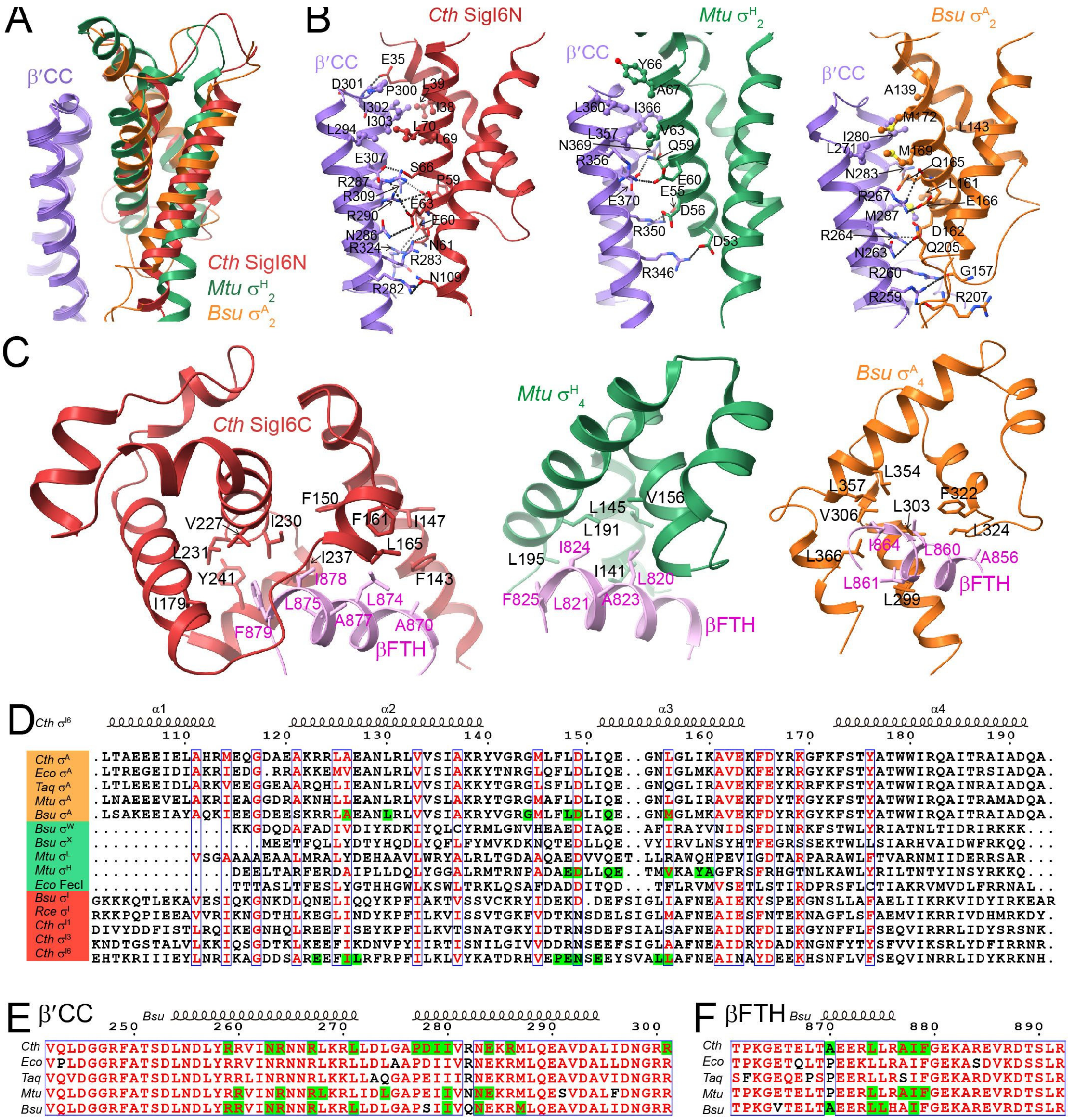

Fig. S5 Interactions between SigI and the RNAP core enzyme in the RPo-SigI6 complex. (A) The structures of β'CC and σ2 in the RPo complexes from different σ^{70} groups. The structures were superimposed by the β'CC regions (purple). The σ2 domains from *Clostridium thermocellum* (*Cth*) SigI6 (PDB no.8I24), *Mycobacterium tuberculosis* (*Mtu*) σ^{H} (PDB no. 5ZX2), and *Bacillus subtilis* (*Bsu*) σ^{A} (PDB no. 7CKQ) are shown in red, green, and brown, respectively. (B) The detailed interactions between the β'CC and σ2 domains. Hydrogen bonds are indicated by black dashed lines. Hydrophobic residues involved in the interaction are shown as balls and sticks. (C) Comparison of the interactions between σ factors and βFTH. The structures of βFTH are shown in magenta. SigI6C and the σ4 domains from *Mycobacterium tuberculosis* σ^{H} and *Bacillus subtilis* σ^{A} are shown in red, green, and brown, respectively. The hydrophobic residues involved in the interactions are shown as sticks. (D) Sequence alignment of σ2 domains from different types of σ^{70} factors. Residues involved in the interactions with RNAP β'CC are highlighted in green. *Eco*, *Escherichia coli*; *Taq*, *Thermus aquaticus*. (E-F) Sequence alignments of RNAP β'CC (E) and βFTH (F) from

different bacterial species. Residues involved in the interactions with σ factors are highlighted in green.

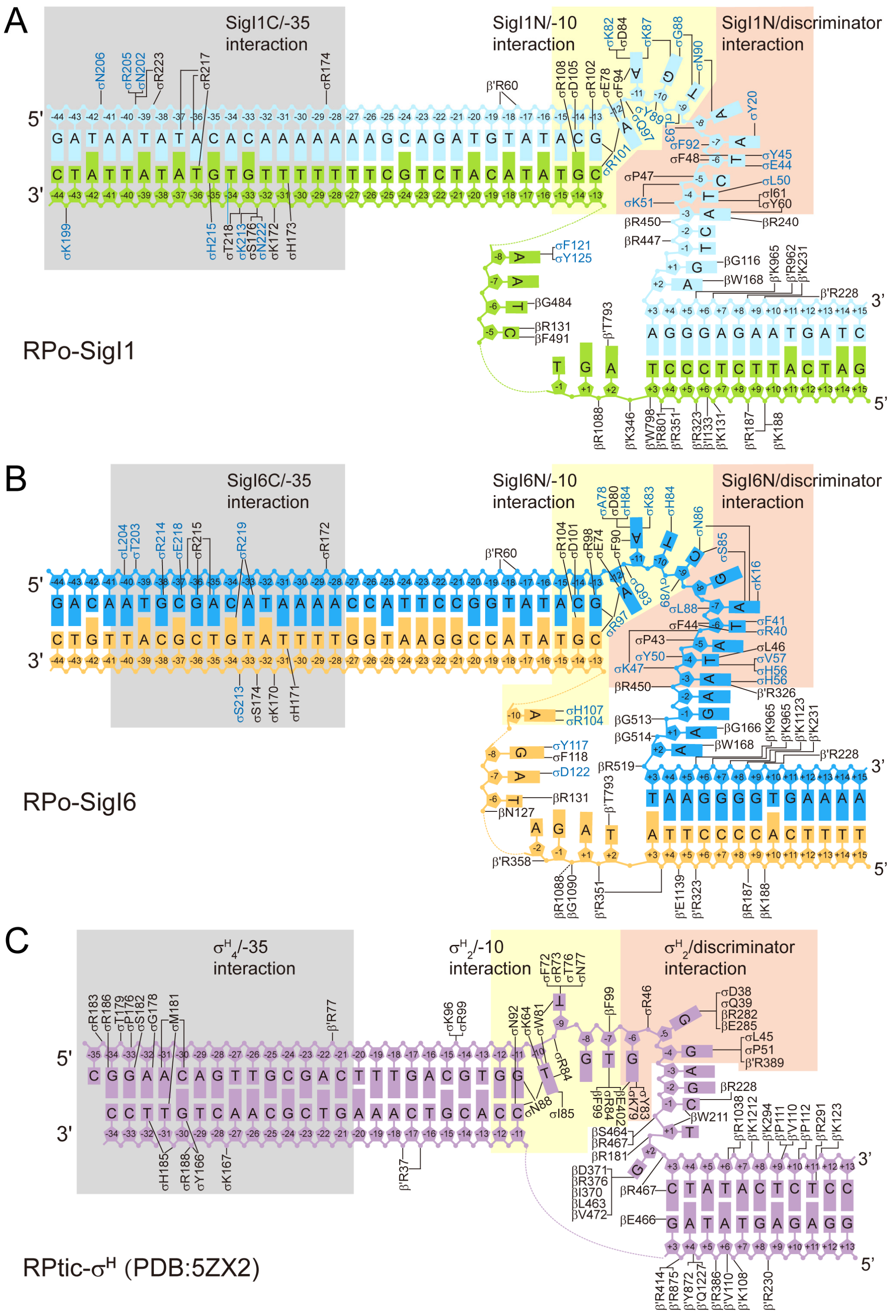

Fig. S6 Protein-DNA interactions in RPo-SigI1, RPo-SigI6, and RPtic-σ^{H} complexes. (A-B) Summary of protein-DNA interactions in RPo-SigI1 (A) and RPo-SigI6 (B). Conserved and non-conserved residues are labeled in black and blue, respectively. (C) Summary of protein-DNA interactions in RPtic-σ^{H} from *M. tuberculosis* (Li *et al.* 2019 *Nature Commun.* 10, 1153).

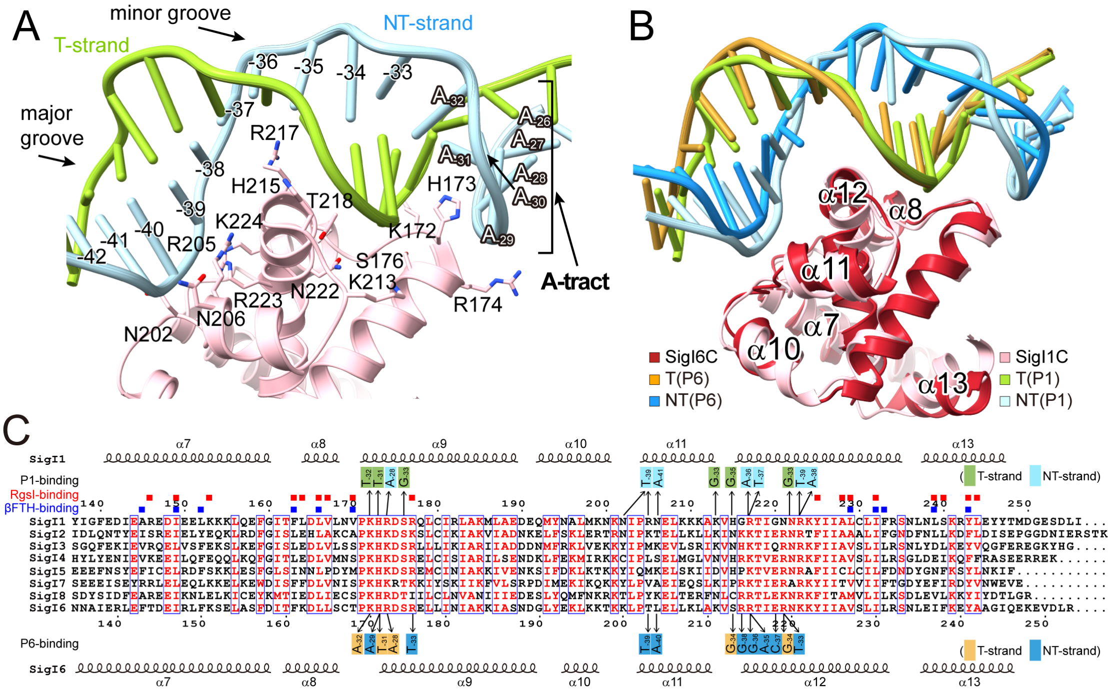

Fig. S7 SigIC-promoter interactions. (A) The interactions between SigI1C and promoter DNA in the RPo-SigI1 structure. Residues involved in the interactions are shown as sticks. (B) Structure comparison of the SigI6C-promoter and SigI1C-promoter in the RPo complexes. The structures were superimposed by the SigIC domains. (C) Sequence alignment of the SigIC domains of eight SigI factors in *C. thermocellum*. The residues involved in promoter interactions are indicated by arrows. Residues interacting with RNAP βFTH and RsgI1N are indicated by blue- and red-filled squares, respectively, at the top of the alignment. The secondary structure elements of SigI1 and SigI6 are shown at the top and at the bottom, respectively.

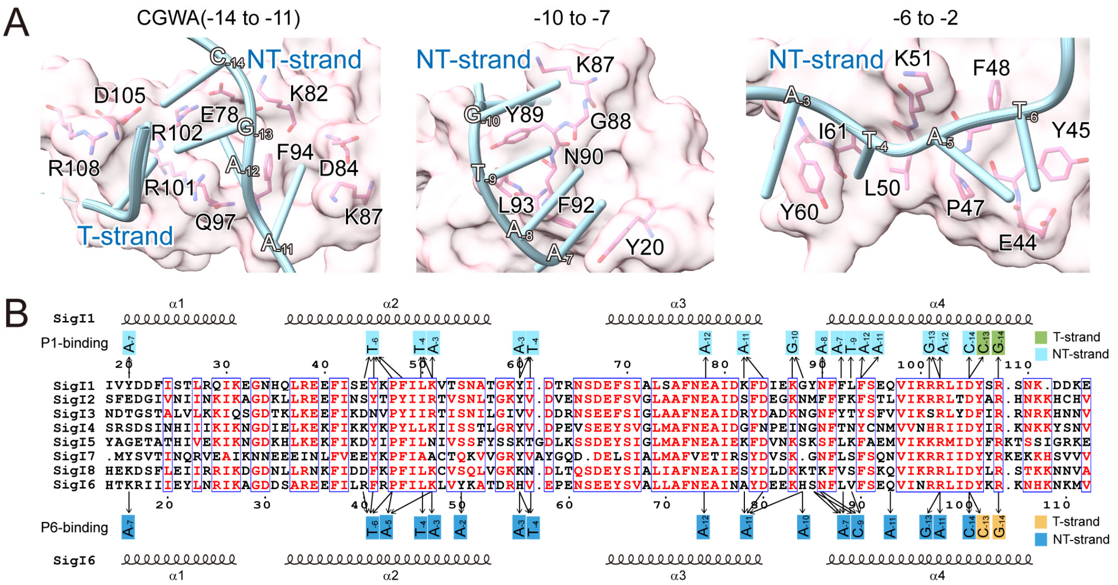

Fig. S8 SigIN-promoter interactions. (A) Detailed interactions between SigI1N and the promoter -10 element DNA of P1. Residues involved in the interactions are shown as sticks. (B) Sequence alignment of the SigIN domains of the eight SigI factors in *C. thermocellum*. The residues involved in promoter interactions are indicated by arrows. The secondary structure elements of SigI1 and SigI6 are shown on the top and at the bottom, respectively.

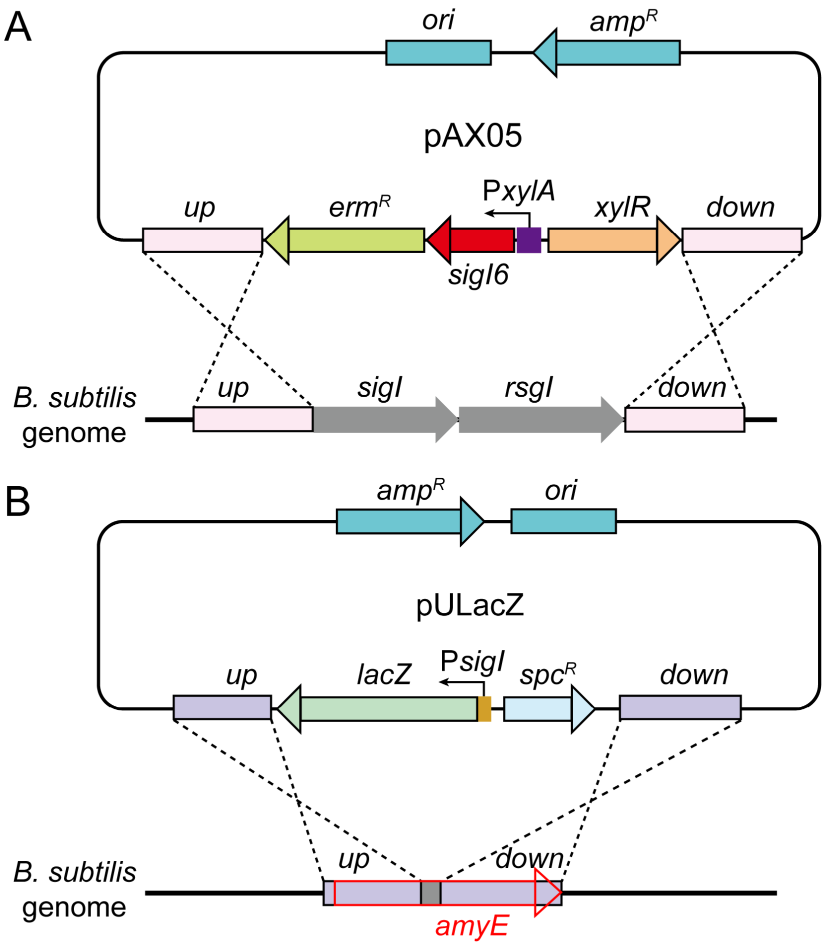

Fig. S9 Schematic diagram of the *B. subtilis* heterologous reporter system. The system contains two parts: a cassette for xylose-induced SigI expression (A) and a cassette of the LacZ reporter with a SigI-dependent promoter (P*sigI*). The simultaneous knock-out of endogenous *sigI-rsgI* genes and knock-in of the cassette for the exogenous xylose-induced *sigI* expression were obtained by homologous recombination. The *E. coli lacZ* gene with a designed SigI-dependent promoter and the spectinomycin resistance gene (*spc*^{R}) were integrated into the *amyE* locus. *up*, upstream homologous region; *down*, downstream homologous region; *amp*^{R}, ampicillin-resistance gene; *erm*^{R}, erythromycin-resistance gene; *spc*^{R}, spectinomycin resistance gene; P*xylA*, xylose-induced promoter; *xylR*, xylose repressor; *amyE*, the integration locus of the promoter reporter system; *ori*, the origin of replication.

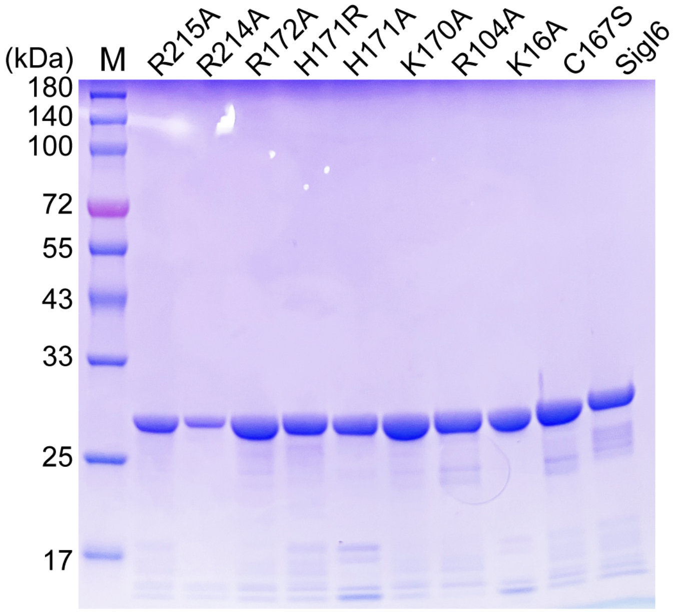

Fig. S10 SDS-PAGE analysis of SigI6 and its mutants used in the *in vitro* transcription assay.

Fig. S1B, left panel

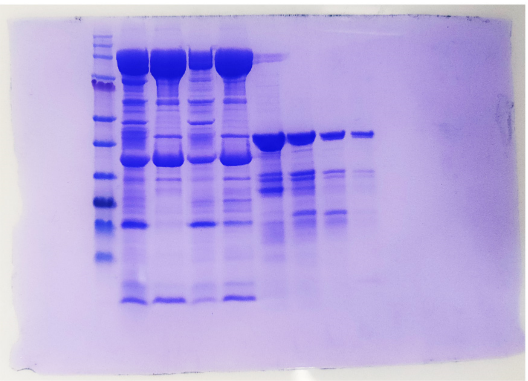

Fig. S1B, right panel

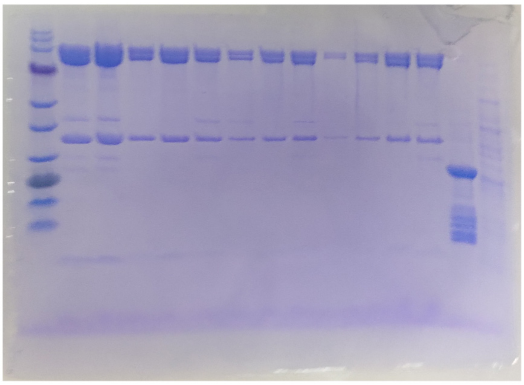

Fig. S1E

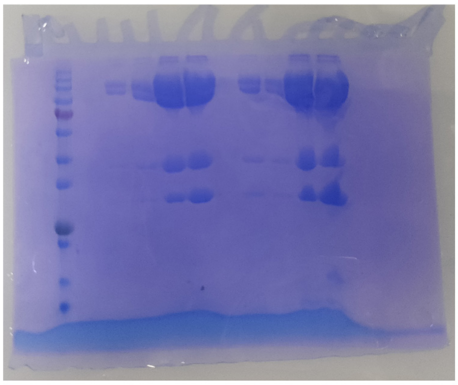

Fig. S1F, left panel

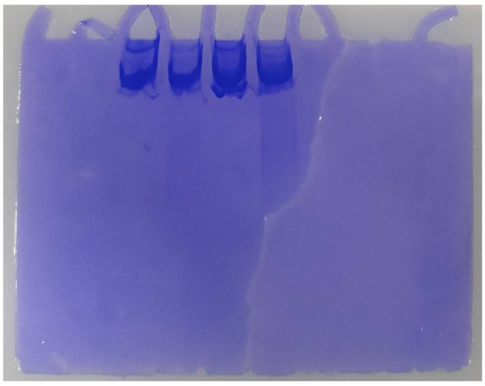

Fig. S1F, right panel

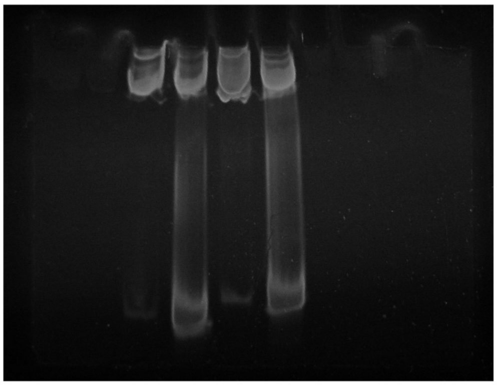

Fig. S10

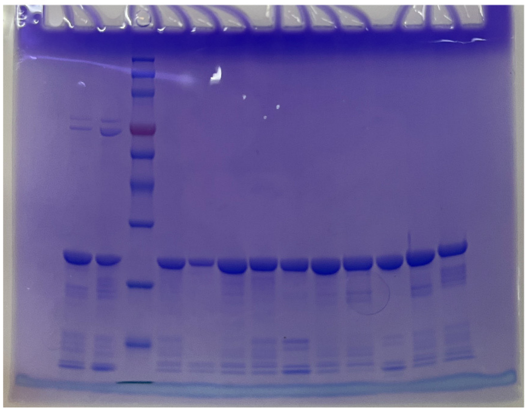

Fig. S11 Original pictures of gels used in this study.

Table S1. Cryo-EM data collection, refinement, and validation statistics.

	RPo-SigI1 (EMDB: EMD-35130) (PDB: 8I23)	RPo-SigI6 (EMDB: EMD-35131) (PDB 8I24)
**Data collection and processing**		
Magnification	22,500	22,500
Voltage (kV)	300	300
Electron exposure (e^{-}/Å^2)	60	60
Defocus range (μm)	1.5-2.5	1.5-2.5
Symmetry imposed	C1	C1
Initial particle images (no.)	2207	5531
Final particle images (no.)	150741	44677
Map resolution (Å)	3.03	3.36
FSC threshold	0.143	0.5
Map resolution range (Å)	2.8-5.5	3.2-4.2
**Refinement**		
Initial model used (PDB code)	N/A	N/A
Model resolution (Å)	3.0	3.3
FSC threshold	0.5	0.5
Map sharpening B factor (Å^2)	-83.85	-113.99
Model composition		
Non-hydrogen atoms	26272	26320
Protein residues	3066	3068
Nucleotides	108	110
Ligands	Zn: 2, Mg: 1	Zn: 2, Mg: 1
B factors (Å^2)		
Protein	42.06	48.59
Nucleotide	84.60	82.84
Ligand	47.58	58.68
R.m.s. deviations		
Bond lengths (Å)	0.003	0.004
Bond angles (°)	0.532	0.605
Validation		
MolProbity score	1.77	1.65
Clashscore	8.84	7.82
Poor rotamers (%)	0.15	0.91
Ramachandran plot		
Favored (%)	95.74	96.59
Allowed (%)	4.26	3.41
Disallowed (%)	0.00	0.00

Table S2. The strains used in this study.

Species	Name	Description
Escherichia coli	TOP10	For plasmid construction.
	BL21::pET28a-SMT3-SigI1	The transformed BL21(DE3) containing the plasmid pET28a-SMT3-SigI1 for overexpression of SigI1.
	BL21::pET28a-SMT3-SigI6-mutants	The transformed BL21(DE3) containing the plasmid pET28a-SMT3-SigI6-mutants for overexpression of mutants of SigI6.
Clostridium thermocellum	DSM1313ΔpyrF	Derived from DSM1313, with deleted *pyrF* gene. Chassis cell for homologous recombination genome editing (Zhang *et al.* 2017 *Biotechnol. Biofuels* 10:124).
	DSM1313::P_{2638}-His10-β′	The strain containing the insertion of the promoter P_{2638} and His×10-tag before the *β′* gene in the genome.
Bacillus subtilis	168	Wide type.
	BC01	168 *sigI-rsgI*::(P*xyl-sigI6*, Erm^R). The intrinsic *sigI-rsgI* of 168 was replaced with the xylose-induced expression cassette of SigI6.
	BC01-PsigI6	168 *sigI-rsgI*::(P*xyl-sigI6*, Erm^R), *amyE*::(P*sigI6-lacZ*, Spc^R). The *amyE* of BC01 was replaced with the LacZ reporter cassette containing the promoter of *sigI6*.
	BC01-PsigI6[Pmut]	168 *sigI-rsgI*::(P*xyl-sigI6*, Erm^R), *amyE*::(P*sigI6*[Pmut]-*lacZ*, Spc^R), in which "Pmut" is one of the following mutants: A-12t/T-10c/T-10g/T-10a/C-9g/G-8c/A-7g/T-6c/A-5c/T-4g/A-3g
	BC02	168 *amyE*::(P*sigI6-lacZ*, Spc^R). The *amyE* of 168 was replaced with the LacZ reporter cassette containing the promoter of *sigI6*.
	BC02-sigI6	168 amyE::(P*sigI6*-lacZ, SpcR), sigI-rsgI::(P*xyl-sigI6*, ErmR). The sigI-rsgI of BC02 was replaced with the Pxyl-sigI6.
	BC02-sigI6[mut]	168 *amyE*::(P*sigI6-lacZ*, Spc^R), *sigI-rsgI*::(P*xyl-sigI6*[mut], Erm^R), in which "mut" is one of the following mutants: C167S/H171A/H171Y/H171F/H171N/H171S/H171R/H171K/K170A/K170R/R172K/R172A/T203A/L204T/K221A/R215A/R214A/R214K/E218A/E218Q/E218R/R219K/R219A/R104A/D101A/R98Q/R97A/R97H/R97S/E74N/E74A/E74Q/F90Y/F90A/Q93V/K83A/D80A/H84A/H84N&S85Y/H84G&S85Y/H84N&S85M/V89A/N86A/N86E/L88A/K16A/K16T/F44A/F41A/R40K/R40E/P43A/V57A/Y50A/K47A/H56A

Table S3 Promoter DNAs used for RPo complex reconstruction.

Promoter		DNA strand (5' to 3')[a]
**P1**	Non-template strand	AGAGCCGATATTAATCGATAATATACACAAAAAAAGCAGATGTATACGAAGTAATCTACTGAAGGGAGAATGATCTGGTG
	Template strand	CACCAGATCATTCTCCCTAGTCATCTAAACTTCGTATACATCTGCTTTTTTTGTGTATATTATCGATTAATATCGGCTCT
**P6**	Non-template strand	GATCCACCTGGGAAGCTGACAATGCGACATAAAACCATTCCGGTATACGAATCGATATAAGAATAAGGGGTGAAATTAAC
	Template strand	GTTAATTTCACCCCTTATAGAATATAGCATTCGTATACCGGAATGGTTTTATGTCGCATTGTCAGCTTCCCAGGTGGATC

[a] The non-complimentary 10-bp nucleotides for the pre-opened bubble are underlined.

Table S4 Promoter DNAs used for *in vitro* transcription assay.

Promoter	DNA strand (5' to 3')[a]
PSigI6-Mango	CTGCACGAACAACGCAAAATGTTTGCAAAATTGGGTCTCAAAT<br>ATCTTGATCCACCTGGGAAGCTGACAATGCGACATAAAACCAT<br>TCCGGTATACGAATCGATATAAGAATAAGGGGTGAAATTAACCG<br>GTGGATTGGCATTTTCAAGGTACGAACGACGACAGGGAACATA<br>CAAAAAGGATTATTATAGGCACGTACGAAGGAAGGATTGGTAT<br>GTGGTATATTCGTACGTGCCGGCCTGCTGGTAATCGCAggccttttttatt<br>taagggcagcttggcgtaatcatggtcatagctgtttcctgtgtg
A-12t-Mango	CTGCACGAACAACGCAAAATGTTTGCAAAATTGGGTCTCAAAT<br>ATCTTGATCCACCTGGGAAGCTGACAATGCGACATAAAACCAT<br>TCCGGTATACGtATCGATATAAGAATAAGGGGTGAAATTAACCG<br>GTGGATTGGCATTTTCAAGGTACGAACGACGACAGGGAACATA<br>CAAAAAGGATTATTATAGGCACGTACGAAGGAAGGATTGGTAT<br>GTGGTATATTCGTACGTGCCGGCCTGCTGGTAATCGCAggccttttttatt<br>taagggcagcttggcgtaatcatg
G-8c-Mango	CTGCACGAACAACGCAAAATGTTTGCAAAATTGGGTCTCAAAT<br>ATCTTGATCCACCTGGGAAGCTGACAATGCGACATAAAACCAT<br>TCCGGTATACGAATCcATATAAGAATAAGGGGTGAAATTAACCG<br>GTGGATTGGCATTTTCAAGGTACGAACGACGACAGGGAACATA<br>CAAAAAGGATTATTATAGGCACGTACGAAGGAAGGATTGGTAT<br>GTGGTATATTCGTACGTGCCGGCCTGCTGGTAATCGCAggccttttttatt<br>taagggcagcttggcgtaatcatg
T-4g-Mango	CTGCACGAACAACGCAAAATGTTTGCAAAATTGGGTCTCAAAT<br>ATCTTGATCCACCTGGGAAGCTGACAATGCGACATAAAACCAT<br>TCCGGTATACGAATCGATAgAAGAATAAGGGGTGAAATTAACCG<br>GTGGATTGGCATTTTCAAGGTACGAACGACGACAGGGAACATA<br>CAAAAAGGATTATTATAGGCACGTACGAAGGAAGGATTGGTAT<br>GTGGTATATTCGTACGTGCCGGCCTGCTGGTAATCGCAggccttttttatt<br>taagggcagcttggcgtaatcatg
A-3g-Mango	CTGCACGAACAACGCAAAATGTTTGCAAAATTGGGTCTCAAAT<br>ATCTTGATCCACCTGGGAAGCTGACAATGCGACATAAAACCAT<br>TCCGGTATACGAATCGATATgAGAATAAGGGGTGAAATTAACCG<br>GTGGATTGGCATTTTCAAGGTACGAACGACGACAGGGAACATA<br>CAAAAAGGATTATTATAGGCACGTACGAAGGAAGGATTGGTAT<br>GTGGTATATTCGTACGTGCCGGCCTGCTGGTAATCGCAggccttttttatt<br>taagggcagcttggcgtaatcatg

[a] The mutation site in each DNA fragment is shown in lowercase red font. Sequence of the Mango riboswitch is underscored. The terminator sequences are shown in lowercase black font.
