# Supplementary material for: Structure of the transcription open complex of distinct σI factors
Source: Nat Commun. 2023 Oct 13;14:6455. doi: 10.1038/s41467-023-41796-4 (PMC10575876; doi:10.1038/s41467-023-41796-4)
Supplement: Supplementary file 3 — Description of Additional Supplementary Files [file 41467_2023_41796_MOESM3_ESM.pdf]

Description of Additional Supplementary Files

Supplementary Data 1: Plasmids used in this study.

Supplementary Data 2: Primers used in this study.
