# Supplementary material for: Structure of the transcription open complex of distinct σI factors
Source: Nat Commun. 2023 Oct 13;14:6455. doi: 10.1038/s41467-023-41796-4 (PMC10575876; doi:10.1038/s41467-023-41796-4)
Supplement: Supplementary file 4 — Supplementary Data 1 [file 41467_2023_41796_MOESM4_ESM.docx]

**Supplementary Data 1. Plasmids used in this study.**

| **Plasmid** | **Description** | **Source** |
| --- | --- | --- |
| pHKm2-homo-5'Betap | To construct the *Clostridium thermocellum* mutant by homologous recombination for RNAP protein purification | This study |
| pAX01 | Plasmid for gene integration and xylose-induced expression in *Bacillus subtilis* | Härtl *et al.* 2001, *J. Bacteriol.* 183, 2696-2699. |
| pAX05 | pAX01 derivative for integration and xylose-induced expression of the *sigI6* gene in *B. subtilis* | This study |
| pULacZ | pUC19 derivative for integration of the *lacZ* gene and P*sigI6* in *B. subtilis* | This study |
| pAXSigI6(mut) | pAX05 derivative for integration and xylose-induced expression of the SigI6 mutant in *B. subtilis*. The “mut” is one of the following mutants:  C167S/H171A/H171Y/H171F/H171N/H171S/H171R/H171K/K170A/K170R/R172K/R172A/T203A/L204T/K221A/R215A/R214A/R214K/E218A/E218Q/E218R/R219K/R219A/R104A/D101A/R98Q/R97A/R97H/R97S/E74N/E74A/E74Q/F90Y/F90A/Q93V/K83A/D80A/H84A/H84N&S85Y/H84G&S85Y/H84N&S85M/V89A/N86A/N86E/L88A/K16A/K16T/F44A/F41A/R40K/R40E/P43A/V57A/Y50A/K47A/H56A | This study |
| pULPSigI6(Pmut) | pULacZ derivative for integration of the *lacZ* gene and P*sigI6* promoter mutant. The “Pmut” is one of the following mutants:  A-12t/T-10c/T-10g/T-10a/C-9g/G-8c/A-7g/T-6c/A-5c/T-4g/A-3g/ | This study |
| pRL277 | A plasmid containing the spectinomycin-resistance gene (*spc^R^*) | Provided by Hui Li |
| pET28a-SMT3-SigI1 | For heterologous expression and purification of SMT3-SigI1 in *E. coli* | Wei *et al.* 2019 *Nucleic Acids Res.* 47, 5988-5997. |
| pET28a-SMT3-SigI6 | For heterologous expression and purification of SMT3-SigI6 in *E. coli* | This study |
| pET28a-SMT3-SigI6-mutants | For heterologous expression and purification of SMT3-SigI6-mutants in *E. coli*. The mutants include the following sites:  C167S/R215A/R214A/H171R/H171A/R172A/K170A/R104A/K16A | This study |
| pUC19-PsigI6-Mango-tR2 | For wild-type transcription template used for the *in vitro* transcription assay | This study |
| pUC19-PsigI6(mut)-Mango-tR2 | pUC19-PsigI6-Mango-tR2 derivative for the *in vitro* transcription assay. The PsigI6(mut) plasmids include one of the following sites: A-12t/G-8c/T-4g/A-3g | This study |
