# Supplementary material for: Structure of the transcription open complex of distinct σI factors
Source: Nat Commun. 2023 Oct 13;14:6455. doi: 10.1038/s41467-023-41796-4 (PMC10575876; doi:10.1038/s41467-023-41796-4)
Supplement: Supplementary file 5 — Supplementary Data 2 [file 41467_2023_41796_MOESM5_ESM.docx]

**Supplementary Data 2. Primers used in this study.**

| Primer name | Sequence (5' to 3')^a^ | Used for |
| --- | --- | --- |
| Bp-UP-F | CTCACTGATTAAGCATTGGTAATCTAGAGAGCCGGTGATATCCTCGTAGG | Construction of plasmid pHKm2-homo-5′Betap |
| Bp-UP-R | GGTATAATATACACTTCCATTCAGGTCGACTTATAAATCATCGTCATCAA |  |
| Bp-DN-F | AAAGTAAGGAGGAATTTGTTCGGCCGATGGGATCATCACATCATCATCATCATCATCATCATCATCATTCAGGATCAGGATCAGGATCAGGATCAGGATTTGAACTTAATAACTTCGA |  |
| Bp-DN-R | GAGCACCGGACATAATCGTACAGATCTGAATTCCTGTCAGACCAAGTTTACTCATATATA |  |
| P2638-F | CGTTGCGAGACAGGAAGTAACTCGAGGATAAACAAAGGACGGTTC |  |
| P2638-R | TGATGATGTGATGATCCCATCGGCCGAACAAATTCCTCCTTACTTTTG |  |
| Test_Plasmid-F1 | TCAGGCAACTATGGATGAAC | Verification of the transformation of plasmid pHKm2-homo-5′Betap in *C. thermocellum* |
| Test_Plasmid-R1 | GCTCAGTGGAACGAAAACTC |  |
| Test_Plasmid-F2 | ACGGGCTCGGAGCAATTGTC |  |
| Test_Plasmid-R2 | GCAAAGTTTTCGGCGGAATG |  |
| Test_Genome-F | CTACGAGGACGCCATCCTGATAAGTG | Verification of strain DSM1313::P*_2638_*-His10-β' |
| Test_Genome-R | CGACCGGAATAGTCAACACGTTTTC |  |
| SigI6-F | CTCACAGAGAACAGATTGGTGGATCCGTGGATTGGCATTTTCAAGGTAC | Construction of plasmids for SigI6 and SigI6-C167S expression in *E. coli* |
| SigI6-R | CAGTGGTGGTGGTGGTGGTGCTCGAGTCACCGCAAATCCACCTCCTTTTC |  |
| SigI6C167S-F | CTTTTAAGGATTTGCTCTCCTcTACTCCAAAGCACAGAGATTC |  |
| SigI6C167S-R | GAATCTCTGTGCTTTGGAGTAgAGGAGAGCAAATCCTTAAAAG |  |
| sigI-F1 | **GCAAATGCAGACAATATCAG**GATCGGGCTGGAAGGGATTTTAG | Construction of plasmid pAX05 |
| sigI-R1 | **GGCAAGAACGTTGCTCTAGA**CTCAGTTCCTCCCTATAACTACTC |  |
| rsgI-F1 | **GCGACTGCAGAGATATCGAT**AGACCTGAATTTATTTAGTTG |  |
| rsgI-R1 | **GCCCTTTCGTCTTCAAGAAT**CGATATGGTGCGGCTGCTCTA |  |
| Bs-sigI6-F1 | **CGGCCGCCCGCGGGAGCTCGGATCC**TCACCGCAAATCCACCTCCTT |  |
| Bs-sigI6-R1 | **ATCAAAGGGGGAAATGGGATCC**GTGGATTGGCATTTTCAAGGTAC |  |
| Bs-P01 | GGAGTAGCGTCCGGAATGTATTC | Verification of the integration of *xylR* and *sigI6* in *B. subtilis* |
| Bs-P09 | GCAATTGCTTAAGCTGCCAGCG |  |
| Bs-P109 | GGAGCGGTTTCTATCGTTATTGATTC |  |
| Bs-P102 | CATAGACCTATATCCGCGTC |  |
| amyE-F1 | **CGCATCTGTGCGGTATTTCAC**ATGGCTGGACAGCCTGAGGAACTC | Construction of plasmid pULacZ |
| amyE-R1 | **CTGAGAGTGCACCATATGCGGT**TCAATGGGGAAGAGAACCGC |  |
| amyE-F2 | **GCCTGCAGGTCGACTCTAGAGGATCC**TAACAAAATTCTCCAG |  |
| amyE-R2 | **GTCGGTTTTCTAATGTCACTGTCGACT**CTTCATCATCATTGGC |  |
| lacZ-F1 | **GTTAAGGGATGCAGGTCGAC**ATGACCATGATTACGGATTCACTGGCCGTCG |  |
| lacZ-R1 | **CAGCAATGGCAAGAACGTCCCGGGGAGCTC**TTATTTTTGACACCAGACCAACTG |  |
| spc-F | **GTTTAATAGTAGGAGTGATAGATT**ATGAGGGAAGCGGTGATCG |  |
| spc-R1 | GTACAGTCGGCATTATCTCATATTATTTGCCGACTACCTTGGTG |  |
| spc-R2 | AGTGACATTAGAAAACCGACTGTAAAAAGTACAGTCGGCATTATCTCAT |  |
| spc-R3 | **GCCAATGATGATGAAGAGTCGAC**AGTGACATTAGAAAACCGAC |  |
| Bs-P110 | GGAAGCGTTCACAGTTTCG | Verification of the integration of *PsigI-lacZ* in *B. subtilis* |
| Bs-P42 | GGCAGCGCAATGACATTCTT |  |
| Bs-P111 | TCCTGGAGCCCGTCAGTATC |  |
| Bs-P112 | CAATGACCACAAGCTCATCT |  |
| C167S-F | CTGTGCTTTGGAGTagaGGAGAGCAAATCCTTAAAAGTTATATC | Construction of the SigI6 mutant plasmid |
| C167S-R | ATTTGCTCTCCtctACTCCAAAGCACAGAGATTCGAGAGAG |  |
| H171A-F | CAAAAGCTCTCTCGAATCTCTtgcCTTTGGAGTACAGGAGAGC |  |
| H171A-R | GCTCTCCTGTACTCCAAAGgcaAGAGATTCGAGAGAGCTTTTG |  |
| H171Y-F | CAAAAGCTCTCTCGAATCTCTgtaCTTTGGAGTACAGGAGAGCAAATC |  |
| H171Y-R | GTACTCCAAAGtacAGAGATTCGAGAGAGCTTTTGATAAATATTG |  |
| H171F-F | CAAAAGCTCTCTCGAATCTCTgaaCTTTGGAGTACAGGAGAGCAAAT |  |
| H171F-R | GTACTCCAAAGttcAGAGATTCGAGAGAGCTTTTGATAAATATTG |  |
| H171N-F | GCTCTCTCGAATCTCTgttCTTTGGAGTACAGGAGAGCAAATC |  |
| H171N-R | GTACTCCAAAGaacAGAGATTCGAGAGAGCTTTTGATAAATATTG |  |
| H171S-F | AGCTCTCTCGAATCTCTagaCTTTGGAGTACAGGAGAGCAAATC |  |
| H171S-R | CTCCAAAGtctAGAGATTCGAGAGAGCTTTTGATAAATATTGC |  |
| H171R-F | AGCTCTCTCGAATCTCTgcgCTTTGGAGTACAGGAGAGCAAATCC |  |
| H171R-R | ACTCCAAAGcgcAGAGATTCGAGAGAGCTTTTGATAAATATTG |  |
| H171K-F | AGCTCTCTCGAATCTCTtttCTTTGGAGTACAGGAGAGCAAATCC |  |
| H171K-R | ACTCCAAAGaaaAGAGATTCGAGAGAGCTTTTGATAAATATTG |  |
| K170A-F | CGAATCTCTGTGagcTGGAGTACAGGAGAGCAAATCCTTAAAAG |  |
| K170A-R | CCTGTACTCCAgctCACAGAGATTCGAGAGAGCTTTTGATAAATATTG |  |
| K170R-F | TCTCGAATCTCTGTGacgTGGAGTACAGGAGAGCAAATCCTTAAAAG |  |
| K170R-R | CTGTACTCCAcgtCACAGAGATTCGAGAGAGCTTTTGATAAATATTGC |  |
| R172A-F | AGCTCTCTCGAATCagcGTGCTTTGGAGTACAGGAGAGCAAATC |  |
| R172A-R | TCCAAAGCACgctGATTCGAGAGAGCTTTTGATAAATATTGC |  |
| R172K-F | AGCTCTCTCGAATCTtTGTGCTTTGGAGTACAGGAGAGCAAATC |  |
| R172K-R | CTCCAAAGCACAaAGATTCGAGAGAGCTTTTGATAAATATTGC |  |
| T203A-F | TCAACAGTTCCAAagcGGGCAACTTTTTGGTTTTTTTTAG |  |
| T203A-R | AGTTGCCCgctTTGGAACTGTTGAAACTGGCAAAAGTTAG |  |
| L204T-F | CAGTTTCAACAGTTCagtTGTGGGCAACTTTTTGGTTTTTTTTAGC |  |
| L204T-R | AGTTGCCCACAactGAACTGTTGAAACTGGCAAAAGTTAGCAGAAG |  |
| K221A-F | CAATTATATATTTagcATTTCTTTCTATAGTCCTTCTG |  |
| K221A-R | ATAGAAAGAAATgctAAATATATAATTGCAGTAAGC |  |
| R215A-F | TATTTTTTATTTCTTTCTATAGTtgcTCTGCTAACTTTTGCCAGT |  |
| R215A-R | ACTGGCAAAAGTTAGCAGAgcaACTATAGAAAGAAATAAAAAAT |  |
| R214A-F | TCTTTCTATAGTCCTagcGCTAACTTTTGCCAGTTTCAACAG |  |
| R214A-R | CAAAAGTTAGCgctAGGACTATAGAAAGAAATAAAAAATATATAATTG |  |
| R214K-F | CTTTCTATAGTCCTTtTGCTAACTTTTGCCAGTTTCAACAGTTC |  |
| R214K-R | CAAAAGTTAGCAaAAGGACTATAGAAAGAAATAAAAAATATATAATTG |  |
| E218A-F | TATTTTTTATTTCTagCTATAGTCCTTCTGCTAACTTTTGC |  |
| E218A-R | GAAGGACTATAGctAGAAATAAAAAATATATAATTGCAGTAAGC |  |
| E218Q-F | TATTTTTTATTTCTttgTATAGTCCTTCTGCTAACTTTTGC |  |
| E218Q-R | GACTATAcaaAGAAATAAAAAATATATAATTGCAGTAAGC |  |
| E218R-F | TATATTTTTTATTTCTgcgTATAGTCCTTCTGCTAACTTTTGCCAG |  |
| E218R-R | TAGCAGAAGGACTATAcgcAGAAATAAAAAATATATAATTGCAGTAAG |  |
| R219K-F | ATATTTTTTATTTtTTTCTATAGTCCTTCTGCTAACTTTTGCCAG |  |
| R219K-R | AGGACTATAGAAAaAAATAAAAAATATATAATTGCAGTAAGC |  |
| R219A-F | ATTTTTTATTagcTTCTATAGTCCTTCTGCTAACTTTTGCCAG |  |
| R219A-R | GCAGAAGGACTATAGAAgctAATAAAAAATATATAATTGCAGTAAGC |  |
| R104A-F | CTTATGATTTTTagcTTTATAGTCAATAAGTCTTCTATTAATAAC |  |
| R104A-R | GACTATAAAgctAAAAATCATAAGAATAAAATGGTTTATCC |  |
| D101A-F | GATTTTTTCTTTTATAagcAATAAGTCTTCTATTAATAACCTGTTCTG |  |
| D101A-R | TAGAAGACTTATTgctTATAAAAGAAAAAATCATAAGAATAAAATGG |  |
| R98Q-F | ATAGTCAATAAGttgTCTATTAATAACCTGTTCTGAGAAAAC |  |
| R98Q-R | GGTTATTAATAGAcaaCTTATTGACTATAAAAGAAAAAATCATAAG |  |
| R97A-F | ATAGTCAATAAGTCTTgcATTAATAACCTGTTCTGAGAAAACAAG |  |
| R97A-R | CAGGTTATTAATgcAAGACTTATTGACTATAAAAGAAAAAATCATAAG |  |
| R97H-F | ATAGTCAATAAGTCTatgATTAATAACCTGTTCTGAGAAAACAAG |  |
| R97H-R | ACAGGTTATTAATcatAGACTTATTGACTATAAAAGAAAAAATCATAAG |  |
| R97S-F | ATAGTCAATAAGTCTTgcATTAATAACCTGTTCTGAGAAAACAAG |  |
| R97S-R | CAGGTTATTAATgcAAGACTTATTGACTATAAAAGAAAAAATCATAAG |  |
| E74N-F | CATAAGCATTGATGGCgttATTGAAAGCCAATAATGCAACGCTG |  |
| E74N-R | TGGCTTTCAATaacGCCATCAATGCTTATGATGAAGAGAAGC |  |
| E74A-F | CTCTTCATCATAAGCATTGATGGCtgcATTGAAAGCCAATAATGCAAC |  |
| E74A-R | GTTGCATTATTGGCTTTCAATgcaGCCATCAATGCTTATGATGAAGAG |  |
| E74Q-F | CATAAGCATTGATGGCttgATTGAAAGCCAATAATGCAACGCTG |  |
| E74Q-R | GCTTTCAATcaaGCCATCAATGCTTATGATGAAGAGAAGCATTC |  |
| F90Y-F | TAACCTGTTCTGAgtaAACAAGGAAGTTAGAATGCTTCTC |  |
| F90Y-R | CTTCCTTGTTtacTCAGAACAGGTTATTAATAGAAGACTTATTG |  |
| F90A-F | TAACCTGTTCTGAagcAACAAGGAAGTTAGAATGCTTCTCTTCATC |  |
| F90A-R | CTAACTTCCTTGTTgctTCAGAACAGGTTATTAATAGAAGACTTATTG |  |
| Q93V-F | CAATAAGTCTTCTATTAATAACtacTTCTGAGAAAACAAGGAAG |  |
| Q93V-R | CTTGTTTTCTCAGAAgtaGTTATTAATAGAAGACTTATTGAC |  |
| K83A-F | AGTTAGAATGagcCTCTTCATCATAAGCATTGATGGCTTCATTG |  |
| K83A-R | GCTTATGATGAAGAGgctCATTCTAACTTCCTTGTTTTCTCAGAAC |  |
| D80A-F | GAATGCTTCTCTTCagcATAAGCATTGATGGCTTCATTGAAAGCC |  |
| D80A-R | TCAATGCTTATgctGAAGAGAAGCATTCTAACTTCCTTGTTTTCTC |  |
| H84A-F | CAAGGAAGTTAGAtgcCTTCTCTTCATCATAAGCATTGATGGCTTC |  |
| H84A-R | CTTATGATGAAGAGAAGgcaTCTAACTTCCTTGTTTTCTCAGAACAG |  |
| H84N&S85G-F | ACAAGGAAGTTaccgttCTTCTCTTCATCATAAGCATTGATGG |  |
| H84N&S85G -R | GATGAAGAGAAGaacggtAACTTCCTTGTTTTCTCAGAACAGG |  |
| H84G&S85Y-F | AACAAGGAAGTTgtaaccCTTCTCTTCATCATAAGCATTGATG |  |
| H84G&S85Y-R | ATGAAGAGAAGggttacAACTTCCTTGTTTTCTCAGAACAGG |  |
| H84N&S85M-F | GGAAGTTcatgttCTTCTCTTCATCATAAGCATTGATGGCTTC |  |
| H84N&S85M-R | TGATGAAGAGAAGaacatgAACTTCCTTGTTTTCTCAGAACAGG |  |
| V89A-F | AACCTGTTCTGAGAAtgCAAGGAAGTTAGAATGCTTCTCTTCATC |  |
| V89A-R | AACTTCCTTGcaTTCTCAGAACAGGTTATTAATAGAAGAC |  |
| N86A-F | GAAAACAAGGAAagcAGAATGCTTCTCTTCATCATAAG |  |
| N86A-R | GAAGCATTCTgctTTCCTTGTTTTCTCAGAACAGG |  |
| N86E-F | AGAAAACAAGGAAttcAGAATGCTTCTCTTCATCATAAG |  |
| N86E-R | AAGCATTCTgaaTTCCTTGTTTTCTCAGAACAGGTTAT |  |
| L88A-F | GTTCTGAGAAAACagcGAAGTTAGAATGCTTCTCTTCATC |  |
| L88A-R | AGCATTCTAACTTCgctGTTTTCTCAGAACAGGTTATT |  |
| K16A-F | CTATAATAATCCTtgcTGTATGTTCCCTGTCGTCGTTCG |  |
| K16A-R | GACAGGGAACATACAgcaAGGATTATTATAGAGTATCTG |  |
| K16T-F | TCTATAATAATCCTagTTGTATGTTCCCTGTCGTCGTTCGTAC |  |
| K16T-R | ACAGGGAACATACAActAGGATTATTATAGAGTATCTGAACAG |  |
| F44A-F | CAATTTTAATATagcAGGCCTAAACCTCAGGATAAACTCTTCC |  |
| F44A-R | GAGGTTTAGGCCTgctATATTAAAATTGGTGTATAAGGCGACTGAC |  |
| F41A-F | TATAAAAGGCCTagcCCTCAGGATAAACTCTTCCCTTGCAG |  |
| F41A-R | TATCCTGAGGgctAGGCCTTTTATATTAAAATTGGTGTATAAGGC |  |
| R40K-F | ATAAAAGGCCTAAATtTCAGGATAAACTCTTCCCTTGC |  |
| R40K-R | TTATCCTGAaATTTAGGCCTTTTATATTAAAATTGGTG |  |
| R40E-F | AAGGCCTAAATtCCAGGATAAACTCTTCCCTTGCAGAATC |  |
| R40E-R | GAAGAGTTTATCCTGGaATTTAGGCCTTTTATATTAAAATTGGTG |  |
| P43A-F | ATTTTAATATAAAtgcCCTAAACCTCAGGATAAACTCTTCCCTTG |  |
| P43A-R | TCCTGAGGTTTAGGgcaTTTATATTAAAATTGGTGTATAAGGCGAC |  |
| V57A-F | CTTCACTGTTTTCCGGCTCagcATGCCTGTCAGTCGCCTTATACACC |  |
| V57A-R | ACAGGCATgctGAGCCGGAAAACAGTGAAGAATACAGCGTTGC |  |
| Y50A-F | CTGTCAGTCGCCTTagcCACCAATTTTAATATAAAAGGCCTAAAC |  |
| Y50A-R | TAAAATTGGTGgctAAGGCGACTGACAGGCATGTTGAGCCG |  |
| K47A-F | CGCCTTATACACCAAagcTAATATAAAAGGCCTAAACCTCAGG |  |
| K47A-R | GCCTTTTATATTAgctTTGGTGTATAAGGCGACTGACAGGCATG |  |
| H56A-F | TCCGGCTCAACagcCCTGTCAGTCGCCTTATACACCAATTTTAAT |  |
| H56A-R | GCGACTGACAGGgctGTTGAGCCGGAAAACAGTGAAGAATACAGC |  |
| A-12t-F | CATTCCGGTATACGtATCGATATAAGAGAAAAGATTAACTAATAAG | Construction of the P*sigI6* promoter mutant plasmid |
| A-12t-R | ATATCGATaCGTATACCGGAATGGTTTTATGTCGCATTGTCAGCTTC |  |
| T-10c-F | TCCGGTATACGAAcCGATATAAGAGAAAAGATTAACTAATAAGGAG |  |
| T-10c-R | TCTCTTATATCGgTTCGTATACCGGAATGGTTTTATGTCGCATTG |  |
| T-10g-F | ATTCCGGTATACGAAgCGATATAAGAGAAAAGATTAACTAATAAG |  |
| T-10g-R | CTTTTCTCTTATATCGcTTCGTATACCGGAATGGTTTTATGTCGCATTG |  |
| T-10a-F | TCCGGTATACGAAaCGATATAAGAGAAAAGATTAACTAATAAG |  |
| T-10a-R | TCTCTTATATCGtTTCGTATACCGGAATGGTTTTATGTCGCATTGTC |  |
| C-9g-F | ATTCCGGTATACGAATgGATATAAGAGAAAAGATTAACTAATAAG |  |
| C-9g-R | TTTCTCTTATATCcATTCGTATACCGGAATGGTTTTATGTCG |  |
| G-8c-F | GTATACGAATCcATATAAGAGAAAAGATTAACTAATAAGGAGGAC |  |
| G-8c-R | ATCTTTTCTCTTATATgGATTCGTATACCGGAATGGTTTTATGTCG |  |
| A-7g-F | GTATACGAATCGgTATAAGAGAAAAGATTAACTAATAAGGAG |  |
| A-7g-R | CTTTTCTCTTATAcCGATTCGTATACCGGAATGGTTTTATGTC |  |
| T-6c-F | CCGGTATACGAATCGAcATAAGAGAAAAGATTAACTAATAAGGAG |  |
| T-6c-R | TCTTTTCTCTTATgTCGATTCGTATACCGGAATGGTTTTATG |  |
| A-5c-F | GTATACGAATCGATcTAAGAGAAAAGATTAACTAATAAGGAGGAC |  |
| A-5c-R | ATCTTTTCTCTTAgATCGATTCGTATACCGGAATGGTTTTATG |  |
| T-4g-F | GTATACGAATCGATAgAAGAGAAAAGATTAACTAATAAGGAGGAC |  |
| T-4g-R | AATCTTTTCTCTTcTATCGATTCGTATACCGGAATGGTTTTATGTC |  |
| A-3g-F | GTATACGAATCGATATgAGAGAAAAGATTAACTAATAAGGAGGAC |  |
| A-3g-R | TAATCTTTTCTCTcATATCGATTCGTATACCGGAATGGTTTTATG |  |
| tR2-FP | CATGATTACGCCAAGCTGCCCTTAAATAAAAAG | Used for amplifying promoter DNA fragments for *in vitro* transcription assay |
| P6-RP | CTGCACGAACAACGCAAAATGTTTG |  |

^a^ The mutation site in each DNA fragment is shown in lowercase red font. Sequences of homology segments for seamless cloning are shown in bold. Restriction sites are underlined.
