# Supplementary material for: Structure of the transcription open complex of distinct σI factors
Source: Nat Commun. 2023 Oct 13;14:6455. doi: 10.1038/s41467-023-41796-4 (PMC10575876; doi:10.1038/s41467-023-41796-4)
Supplement: Supplementary file 6 — Reporting Summary [file 41467_2023_41796_MOESM6_ESM.pdf]

nature portfolio

Corresponding author(s): Yingang Feng

Last updated by author(s): Sep 5, 2023

Reporting Summary

Nature Portfolio wishes to improve the reproducibility of the work that we publish. This form provides structure for consistency and transparency in reporting. For further information on Nature Portfolio policies, see our Editorial Policies and the Editorial Policy Checklist.

Statistics

For all statistical analyses, confirm that the following items are present in the figure legend, table legend, main text, or Methods section.

n/a	Confirmed	
☐	☒	The exact sample size (*n*) for each experimental group/condition, given as a discrete number and unit of measurement
☐	☒	A statement on whether measurements were taken from distinct samples or whether the same sample was measured repeatedly
☒	☐	The statistical test(s) used AND whether they are one- or two-sided<br>*Only common tests should be described solely by name; describe more complex techniques in the Methods section.*
☒	☐	A description of all covariates tested
☒	☐	A description of any assumptions or corrections, such as tests of normality and adjustment for multiple comparisons
☒	☐	A full description of the statistical parameters including central tendency (e.g. means) or other basic estimates (e.g. regression coefficient) AND variation (e.g. standard deviation) or associated estimates of uncertainty (e.g. confidence intervals)
☒	☐	For null hypothesis testing, the test statistic (e.g. *F*, *t*, *r*) with confidence intervals, effect sizes, degrees of freedom and *P* value noted<br>*Give P values as exact values whenever suitable.*
☒	☐	For Bayesian analysis, information on the choice of priors and Markov chain Monte Carlo settings
☒	☐	For hierarchical and complex designs, identification of the appropriate level for tests and full reporting of outcomes
☒	☐	Estimates of effect sizes (e.g. Cohen's *d*, Pearson's *r*), indicating how they were calculated

Our web collection on statistics for biologists contains articles on many of the points above.

Software and code

Policy information about availability of computer code

Data collection	SerialEM
Data analysis	Warp 1.0.9 , Relion 3.0, CryoSPARC v3.3, CryoDRGN 1.0, PHENIX 1.19.2, Chimera 1.16, ChimeraX 1.3, PyMOL 2.5

For manuscripts utilizing custom algorithms or software that are central to the research but not yet described in published literature, software must be made available to editors and reviewers. We strongly encourage code deposition in a community repository (e.g. GitHub). See the Nature Portfolio guidelines for submitting code & software for further information.

Data

Policy information about availability of data

All manuscripts must include a data availability statement. This statement should provide the following information, where applicable:

- Accession codes, unique identifiers, or web links for publicly available datasets
- A description of any restrictions on data availability
- For clinical datasets or third party data, please ensure that the statement adheres to our policy

The data needed to evaluate the conclusions of the paper are present in the paper and/or the Supplementary information. Source data are provided as a Source Data file. The electron density maps of RPo-SigI1 and RPo-SigI6 are deposited in the Electron Microscopy Data Bank (https://www.ebi.ac.uk/pdbe/emdb/) under accession numbers EMD-35130 and EMD-35131, respectively. The atomic models of RPo-SigI1 and RPo-SigI6 have been deposited to the Protein Data Bank (https://www.rcsb.og/) under accession codes 8I23 and 8I24, respectively. Other structure data used in this study for analysis (7MKP [https://www.rcsb.org/structure/7MKP], 6CA0 [https://www.rcsb.org/structure/6CA0], 7CKQ [https://www.rcsb.org/structure/7CKQ], 6MPJ [https://www.rcsb.org/structure/

unreleased/6MPJ], 5ZX2 [https://www.rcsb.org/structure/5ZX2], 6IVU [https://www.rcsb.org/structure/6IVU]) are available in the Protein Data Bank.

Research involving human participants, their data, or biological material

Policy information about studies with human participants or human data. See also policy information about sex, gender (identity/presentation), and sexual orientation and race, ethnicity and racism.

Reporting on sex and gender	This is not relevant for this study
Reporting on race, ethnicity, or other socially relevant groupings	This is not relevant for this study
Population characteristics	This is not relevant for this study
Recruitment	This is not relevant for this study
Ethics oversight	This is not relevant for this study

Note that full information on the approval of the study protocol must also be provided in the manuscript.

Field-specific reporting

Please select the one below that is the best fit for your research. If you are not sure, read the appropriate sections before making your selection.

[x] Life sciences [ ] Behavioural & social sciences [ ] Ecological, evolutionary & environmental sciences

For a reference copy of the document with all sections, see nature.com/documents/nr-reporting-summary-flat.pdf

Life sciences study design

All studies must disclose on these points even when the disclosure is negative.

Sample size	For each RPo complex, one cryo-EM dataset comprising several thousand micrographs was collected. The number of micrographs in each dataset was determined based on our experience and the available microscope time.
Data exclusions	No data were excluded.
Replication	Each Cryo-EM dataset was collected in one session one replicate with hundreds of thousands particles . Activity experiments are replicated three times. We confirmed all attempts at replication were successful.
Randomization	Random particle subsets were used during the 3D auto-refinement steps in Relion/cryoSPARC and for calculation of Rosenthal-Henderson B-factor plots.
Blinding	No blinding experiments are included. An individual person conducted each experiment according to their expertise.

Reporting for specific materials, systems and methods

We require information from authors about some types of materials, experimental systems and methods used in many studies. Here, indicate whether each material, system or method listed is relevant to your study. If you are not sure if a list item applies to your research, read the appropriate section before selecting a response.

Materials & experimental systems

n/a	Involved in the study	
[x]	[ ]	Antibodies
[x]	[ ]	Eukaryotic cell lines
[x]	[ ]	Palaeontology and archaeology
[x]	[ ]	Animals and other organisms
[x]	[ ]	Clinical data
[x]	[ ]	Dual use research of concern
[x]	[ ]	Plants

Methods

n/a	Involved in the study	
[x]	[ ]	ChIP-seq
[x]	[ ]	Flow cytometry
[x]	[ ]	MRI-based neuroimaging
